# Predictors of non-invasive therapy and 28-day-case fatality in elderly compared to younger patients with acute myocardial infarction: an observational study from the MONICA/KORA Myocardial Infarction Registry

**DOI:** 10.1186/s12872-016-0322-3

**Published:** 2016-07-13

**Authors:** Ute Amann, Inge Kirchberger, Margit Heier, Christian Thilo, Bernhard Kuch, Annette Peters, Christa Meisinger

**Affiliations:** MONICA/KORA Myocardial Infarction Registry, Central Hospital of Augsburg, Stenglinstr. 2, 86156 Augsburg, Germany; Institute of Epidemiology II, Helmholtz Zentrum München, German Research Center for Environmental Health (GmbH), Neuherberg, Germany; Department of Internal Medicine I - Cardiology, Central Hospital of Augsburg, Augsburg, Germany; Department of Internal Medicine/Cardiology, Hospital of Nördlingen, Nördlingen, Germany

**Keywords:** Myocardial infarction, Mortality, Invasive therapy, Predictors

## Abstract

**Background:**

A substantial proportion of patients with acute myocardial infarction (AMI) did not receive invasive therapy, defined as percutaneous coronary intervention and/or coronary artery bypass grafting. Aims of this study were to evaluate predictors of non-invasive therapy in elderly compared to younger AMI patients and to assess the association between invasive therapy and 28-day-case fatality.

**Methods:**

From the German population-based registry, 3475 persons, consecutively hospitalized with an AMI between 2009 and 2012 were included. Data were collected by standardized interviews and chart review. All-cause mortality was assessed on a regular basis. Multivariable logistic regression analyses were conducted.

**Results:**

The sample consisted of 1329 patients aged 28–65 years (age category [AC] 1), 1083 aged 65–74 years (AC 2), and 1063 aged 75–84 years (AC 3). The proportion of patients receiving non-invasive therapy was 10.7, 17.7, and 35.8 % in AC 1, 2, and 3, respectively. Predictors of non-invasive therapy in all ACs were non-ST segment elevation MI, bundle branch block, reduced left ventricular ejection fraction, prior stroke, absence of hyperlipidemia, and low creatine kinase. Elderly women (≥65 years) were less likely to receive invasive therapy. Stratifying the models by type of AMI revealed fewer predictors in patients with ST segment elevation MI. Regarding 28-day-case fatality, strong inverse relations with invasive therapy were seen in all AC: odds ratio of 0.35 (95 % confidence interval [CI] 0.15–0.84), 0.45 (95 % CI 0.22–0.92), and 0.39 (95 % CI 0.24–0.63) in AC 1, 2 and 3, respectively.

**Conclusion:**

In today’s real-life patient care we found that predictors of non-invasive therapy were predominantly the same in all age groups, but differed particularly by type of AMI. Further research is necessary to investigate the real reasons for non-invasive therapy, especially among elderly women. Moreover, we confirmed that receiving invasive therapy was inversely associated with 28-day-case fatality independent of age.

## Background

Percutaneous coronary intervention (PCI) and coronary artery bypass grafting (CABG) are today’s standard invasive treatment options for patients with acute coronary syndrome (ACS) independent of patient’s chronological age [1–5]. Over the last years, an increasing trend in use of these invasive procedures in patients with an acute myocardial infarction (AMI) was reported in several registry studies [6–9]. Nevertheless, there is still a substantial proportion of AMI patients who neither receive PCI nor CABG, even though being eligible for invasive therapy. Earlier studies have determined reasons for the underuse of reperfusion therapy in ST-segment elevation myocardial infarction (STEMI) patients and found that several factors such as older age, female sex, delayed presentation, comorbidities, prior stroke, prior MI, contraindications to the use of fibrinolytic agents and/or mechanical reperfusion (e.g., bleeding risk) are related with no reperfusion therapy in the acute setting [7, 9–13]. To our knowledge, previous studies have not investigated predictive factors of invasive treatment in patients with non-ST segment elevation myocardial infarction (NSTEMI) and did not distinct between younger and elderly persons beneath consideration of short-term survival. The aim of this study was firstly to evaluate predictive factors for non-invasive treatment in elderly and younger AMI patients including the type of AMI (e.g., STEMI, NSTEMI). Secondly, to assess the association between invasive compared to non-invasive therapy and 28-day-case fatality by age group in real-life patient care.

## Methods

### Study design and data source

The present study is based on data from the population-based MI registry in Augsburg, Germany, which was established in 1984 as part of the World Health Organization MONICA Project (MONItoring Trends and Determinants in CArdiovascular disease). After the termination of MONICA in 1995, the MI registry became part of the framework of KORA (Cooperative Health Research in the Region of Augsburg). Since 1984, coronary deaths and non-fatal (at least 24 h surviving) AMI cases of the 25- to 74-year old inhabitants of the city of Augsburg and 2 adjacent counties (about 600,000 inhabitants) have been continuously registered. About 80 % of all AMI cases of the study region are treated in the region’s major hospital, Klinikum Augsburg, a tertiary care center offering 24/7 interventional cardiovascular procedures, as well as heart surgery facilities. From 2009 onwards, the registry was extended for the elderly up to 84 years. The methods of case identification, diagnostic classification of events, and data quality control have been described in detail elsewhere [14, 15]. Since 2001, diagnostic criteria according to the European Society of Cardiology and American College of Cardiology criteria were used for case identification, including assessment of troponin levels especially for identification of NSTEMI [16].

### Data collection

Patients were interviewed during hospital stay by trained nurses using a standardized questionnaire to collect sociodemographic characteristics, cardiovascular risk factors, medical history of previous MI, stroke and comorbidities, and information on the acute event. Further information on type of AMI, treatment procedures and complications during hospital stay, vital signs, medical history, and medication use during hospitalization were collected by review of medical chart. Information provided by the patient concerning the medical history had to be confirmed by chart review. Information on renal dysfunction was collected by review of medical chart. Data collection of the MONICA/KORA MI registry has been approved by the ethics committee of the Bavarian Medical Association (Bayerische Landesärztekammer) and all study participants gave written informed consent.

### Study population

The present study included all consecutive patients aged 25–84 years, who were hospitalized with a non-fatal AMI between January 1, 2009, and December, 31, 2012 and survived longer than 24 h. From 3669 persons, we excluded 194 (5.3 %) individuals with missing information on any of the relevant covariables. Thus, the final study population covered 3475 cases (2397 males and 1078 females) with AMI. Excluded patients due to missing covariable information were older (median age 72 vs. 69 years, p <0.0001), had more frequently a NSTEMI (67.5 vs. 53.3 %) or bundle branch block (BBB) (12.4 vs. 9.5 %, p <0.0001), were less likely to receive an invasive therapy (52.1 vs. 79.5 %, p <0.0001) and coronary angiography (61.9 vs. 89.2 %, p <0.0001), and showed a higher rate of 28-day-case fatality (23.7 vs. 7.4 %, p <0.0001) compared to patients included in the study population.

### Definitions and outcome

The following 3 age categories (AC) were analyzed: AC 1) patients aged 25–64 years, AC 2) patients aged 65–74 years, and AC 3) patients aged 75–84 years. Patients were grouped regarding their in-hospital treatment strategy: invasive therapy was defined as PCI with stent implantation or balloon dilatation or/and CABG; non-invasive (conservative) therapy included patients treated with thrombolysis and/or receiving coronary angiography but without a treatment procedure. The type of AMI was defined as STEMI, NSTEMI, or BBB. The BBB group contains newly developed left BBB, right BBB, and pre-known BBB. Because we do not exactly know whether all patients with a BBB had a newly developed left BBB, which is considered as STEMI equivalent, we displayed the BBB group as separate category.

The outcome of this study was 28-day-case fatality after AMI. Mortality was assessed by checking the vital status of all registered persons of the MONICA/KORA MI registry on a regular basis. Death certificates were obtained from local health departments.

### Data analysis

Categorical variables were expressed as absolute numbers and percentages (%), continuous variables as median with interquartile range (25th and 75th percentiles). For descriptive purpose, the 3 ACs were compared using Chi^2^-test or Fisher’s exact test for categorical variables and the Kruskal-Wallis test (Wilcoxon Analysis) for continuous variables.

To identify predictors that determined the selection of in-hospital treatment strategy (invasive or conservative therapy), multivariable logistic regression analyses were performed for each AC and further stratified by type of AMI. Variables analyzed as potential predictive factors were sex (male/female), smoking (current smoker/ex-smoker/never-smoker/missing), living alone (yes/no/missing), body mass index ≥30 kg/m^2^ (yes/no), prior MI, prior stroke, medical history of diabetes, hyperlipidemia, hypertension, angina pectoris, and chronic obstructive pulmonary disease (yes/no), renal dysfunction reported in medical chart (yes/no), pre-hospital time (symptom onset to arrival) [min] (continuous), left ventricular ejection fraction (LVEF) (> 30 %/≤ 30 %/not assessed or missing), type of AMI (STEMI/NSTEMI/BBB), and peak serum creatine phosphokinase (CPK) level (U/l) during hospitalization (continuous). As criterion for entry in the models, the explanatory variables must meet the 0.05 significance level in at least 1 AC in the bivariate analysis with the in-hospital treatment strategy. The models of the sub-samples by type of AMI included only factors which significantly (*p* < 0.05) contributed to the model using forward selection technique.

To investigate the associations between in-hospital treatment strategy and 28-day-case fatality, further multivariable logistic regression models for each AC were performed. In addition to the above mentioned variables, the following potential confounding factors were considered: in-hospital cardiac arrest (yes/no), any other in-hospital complication (cardiogenic shock or ventricular fibrillation or ventricular tachycardia or recurrent infarction or pulmonary edema or bradycardia [heart rate <50/min] or stroke or any major bleeding complication [intracranial or retroperitoneal or any other major spontaneous bleeding]) (yes/no), married (yes/no/missing), and use of the following evidence-based medication regarded as cornerstone of long-term medical therapy in ACS patients [1–5]: dual antiplatelet therapy, beta-blockers, angiotensin-converting-enzyme inhibitors or angiotensin receptor blockers (ACEIs/ARBs), and statins. We considered a model for each AC adjusted for sex and a full model with additional adjustment for all bivariately significant (*p* < 0.05) variables. A forward stepwise selection technique was used. Variables with more than 2 characteristic (e.g., yes/no/missing) were ‘dummy’-coded.

All analyses were performed using SAS version 9.2 (SAS Institute Inc., Cary, North Carolina).

## Results

The study population consisted of 3475 patients (69.0 % men) with a median age of 69.0 years (interquartile range 58–76 years). There were 1329 (38.2 %) patients in AC 1, 1083 (31.2 %) in AC 2, and 1063 (30.6 %) in AC 3. Baseline characteristics, in-hospital procedures and in-hospital medications according to ACs are shown in Tables 1 and 2. The 3 ACs significantly differed from each other in terms of the analyzed variables except for pre-hospital time and the use of at least 1 antiplatelet agent. The proportion of patients not receiving an invasive therapy was 10.7, 17.7, and 35.8 % in AC 1, 2, and 3; in the sub-group of STEMI patients it was 3.4, 7.6, and 16.4 %, respectively. In general, patients with NSTEMI or BBB were less likely to receive coronary angiography, invasive therapy and the evidence-based medication (except for beta-blockers) compared with STEMI patients (Table 2). The highest proportion of patients treated conservatively was observed in AC 3 in patients with BBB (47.6 %) and with NSTEMI (41.9 %); both sub-groups showed the lowest rate of angiography of 71.7 and 74.0 %, respectively (Table 2).

**Table 1:** Baseline characteristics of the study population by age category (*n* = 3475)

	Age category			
Variable	25–64 years *n* = 1329 (38.2)	65–74 years *n* = 1083 (31.2)	75–84 years *n* = 1063 (30.6)	*p* Value
Sociodemographics				
Age (years)^a^	55 (49–60)	70 (68–72)	79 (77–81)	<0.0001
Female sex	251 (18.9)	327 (30.2)	500 (47.0)	<0.0001
Married^b^	885 (69.6)	777 (75.6)	607 (61.9)	<0.0001
Living alone^b^	255 (20.1)	189 (18.4)	278 (28.4)	<0.0001
Body mass index ≥30 kg/m^2^	413 (31.1)	255 (23.6)	183 (17.2)	<0.0001
Smoker				<0.0001
Current smoker	714 (53.7)	201 (18.6)	74 (7.0)	
Ex-smoker	337 (25.4)	403 (37.2)	308 (29.0)	
Never-smoker	234 (17.6)	391 (36.1)	476 (44.8)	
missing/not known	44 (3.3)	88 (8.1)	205 (19.3)	
Medical history^c^				
Prior MI	181 (13.6)	228 (21.1)	244 (23.0)	<0.0001
Prior stroke	51 (3.8)	117 (10.8)	171 (16.1)	<0.0001
Diabetes	353 (26.6)	439 (40.5)	439 (41.3)	<0.0001
Hypertension	910 (68.5)	885 (81.7)	943 (88.7)	<0.0001
Hyperlipidemia	631 (47.5)	538 (49.7)	453 (42.6)	0.004
Angina pectoris	135 (10.2)	187 (17.3)	210 (19.8)	<0.0001
Chronic obstructive pulmonary disease	50 (3.8)	92 (8.5)	92 (8.7)	<0.0001
Clinical characteristics				
Type of AMI				<0.0001
STEMI	625 (47.0)	369 (34.1)	298 (28.0)	
NSTEM	651 (49.0)	603 (55.7)	599 (56.4)	
Bundle branch block	53 (4.0)	111 (10.2)	166 (15.6)	
Peak serum CPK level (U/l)^a,b^	744 (265–1915)	530 (212–1292)	395 (173–991)	<0.0001
LVEF				<0.0001
> 30 %	1141 (85.8)	841 (77.7)	711 (67.0)	
≤ 30 %	73 (5.5)	87 (8.0)	123 (11.5)	
not assessed/missing	115 (8.7)	155 (14.3)	229 (21.5)	
Renal dysfunction^d^	54 (4.1)	159 (14.7)	306 (28.8)	<0.0001
Pre-hospital time/symptom onset to arrival (min)^a^	159 (79–585)	175 (77–613)	188 (75–565)	0.95

Data are presented as number (percentage) unless otherwise indicated. *P* values were calculated for comparison of the age categories

*AMI* acute myocardial infarction, *STEMI* ST-elevation myocardial infarction, *NSTEMI* non-ST-elevation myocardial infarction, *CPK* creatine phosphokinase, *LVEF* left ventricular ejection fraction

^a^ Presented as median values (25th, 75th percentiles)

^b^ Values were calculated without patients with missing data regarding married (*n* = 195), living alone status (*n* = 195), and peak serum CPK level (*n* = 23)

^c^ Patient-reported medical history of known comorbidities before the acute event, which was collected with a standardized interview during hospital stay and further data were gathered in a concluding chart review. If the information on comorbidities from patient-report and medical chart differed, the chart information was used

^d^ Information on renal dysfunction was collected by review of medical chart

**Table 2:** In-hospital procedures and treatment strategy by age category and stratified by type of AMI (*n* = 3475)

	Age category									
Variable	25–64 years *n* = 1329 (38.2)			65–74 years *n* = 1083 (31.2)			75–84 years *n* = 1063 (30.6)			*p* Value
Type of AMI	STEMI *n* = 625 (47.0)	NSTEMI *n* = 651 (49.0)	BBB *n* = 53 (4.0)	STEMI *n* = 369 (34.1)	NSTEMI *n* = 603 (55.7)	BBB *n* = 111 (10.2)	STEMI *n* = 298 (28.0)	NSTEMI *n* = 599 (56.4)	BBB *n* = 166 (15.6)	<0.0001
Diagnostic procedure										
Coronary angiography	622 (99.5)	606 (93.1)	48 (90.6)	361 (97.8)	534 (88.6)	94 (84.7)	272 (91.3)	443 (74.0)	119 (71.7)	0.003
Treatment strategy										<0.0001
PCI	554 (88.6)	460 (70.7)	31 (58.4)	295 (79.9)	346 (57.4)	69 (62.2)	215 (72.2)	272 (45.4)	72 (43.4)	
CABG	49 (7.8)	84 (12.9)	9 (17.0)	46 (12.5)	118 (19.6)	17 (15.3)	33 (11.1)	76 (12.7)	15 (9.0)	
No invasive therapy^a^	21 (3.4)	106 (16.3)	13 (24.5)	28 (7.6)	138 (22.9)	24 (21.6)	49 (16.4)	251 (41.9)	79 (47.6)	
Thrombolysis	1 (0.2)	1 (0.2)	0	0	1 (0.2)	1 (0.9)	1 (0.3)	0	0	
Evidence-based medication										
Antiplatelet agents	623 (99.7)	643 (98.8)	52 (98.1)	367 (99.5)	600 (99.5)	109 (98.2)	295 (99.0)	590 (98.5)	164 (98.8)	0.24
DAPT	587 (93.9)	543 (83.4)	44 (83.0)	329 (89.2)	459 (76.1)	82 (73.9)	253 (84.9)	404 (67.5)	117 (70.5)	<0.0001
Beta-blockers	601 (96.2)	635 (97.5)	50 (94.3)	351 (95.1)	581 (96.4)	105 (94.6)	274 (92.0)	565 (94.3)	155 (93.4)	0.0006
Statins	596 (95.4)	612 (94.0)	48 (90.6)	346 (93.8)	562 (93.2)	94 (84.7)	270 (90.6)	521 (87.0)	147 (88.6)	<0.0001
ACEIs/ARBs	587 (93.9)	584 (89.7)	48 (90.6)	337 (91.3)	544 (90.2)	96 (86.5)	255 (85.6)	507 (84.6)	139 (83.7)	<0.0001

Data are presented as number (percentage). *P* values were calculated for comparison of the age categories. Data of the total sample of each age category are not presented in this table, but used for comparison tests

*AMI* acute myocardial infarction, *STEMI* ST-elevation myocardial infarction, *NSTEMI* non-ST-elevation myocardial infarction, *BBB* bundle branch block, *PCI* percutaneous coronary intervention, *CABG* coronary artery bypass graft, *DAPT* dual antiplatelet therapy, *ACEIs*/*ARBs* angiotensin-converting enzyme inhibitors or angiotensin-receptor blockers

^a^ Invasive therapy was defined as PCI with stent implantation or balloon dilatation or/and CABG

### Factors associated with in-hospital treatment strategy

The multivariable logistic regression models revealed that type of AMI (NSTEMI vs. STEMI and BBB vs. STEMI), LVEF (LVEF ≤30 vs. LVEF >30 % and LVEF not assessed or missing vs. LVEF >30 %), prior stroke, no hyperlipidemia, and low peak CPK level were strong predictors of non-invasive therapy in all ACs (Table 3). Being a woman was a significant predictor in AC 2 (odds ratio [OR] 1.80; 95 % confidence interval [CI] 1.24–2.61) and AC 3 (OR 1.38; 95 % CI 1.03–1.85); whereas renal dysfunction and prior MI was significantly associated with non-invasive therapy in AC 1 and 2. Stratifying the models by type of AMI showed more differentiated results. For example, in patients with STEMI 3 factors were associated with non-invasive therapy: prior stroke in AC 1 (OR 11.2; 95 % CI 2.58–48.7), female sex in AC 2 (OR 2.35; 95 % CI 1.06–5.21), and LVEF (reduced LVEF and/or LVEF not assessed/missing) in all ACs. In patients with NSTEMI, LVEF was seen as predictor in all ACs, whereas patients with renal dysfunction, prior MI and history of chronic obstructive pulmonary disease demonstrated higher odds of receiving conservative therapy in AC 1 and 2. Prior stroke was observed as strong predictor in AC 3 with NSTEMI (Table 3).

**Table 3:** Factors associated with non-invasive therapy by age category for the total sample and stratified by type of AMI

	Age category					
	25–64 years		65–74 years		75–84 years	
	OR [95 % CI]	*p* Value	OR [95 % CI]	*p* Value	OR [95 % CI]	*p* Value
Total sample (*n* = 3452)^a^	(*n* = 1324)		(*n* = 1077)		(*n* = 1051)	
Sex (women vs. men)	1.28 [0.80–2.06]	0.30	1.80 [1.24–2.61]	0.002	1.38 [1.03–1.85]	0.03
Type of AMI (NSTEMI vs. STEMI)	3.07 [1.81–5.20]	<0.0001	2.54 [1.59–4.05]	<0.0001	2.36 [1.59–3.51]	<0.0001
Type of AMI (BBB vs. STEMI)	4.22 [1.76–10.1]	0.001	2.01 [1.05–3.84]	0.03	2.60 [1.58–4.26]	0.001
Prior stroke (yes vs. no)	2.97 [1.40–6.30]	0.005	1.91 [1.17–3.13]	0.01	1.87 [1.27–2.75]	0.002
Renal dysfunction (yes vs. no)	2.27 [1.06–4.90]	0.04	1.59 [1.03–2.45]	0.04	1.35 [0.97–1.87]	0.07
Prior MI (yes vs. no)	2.08 [1.27–3.41]	0.004	2.00 [1.35–2.95]	0.001	1.27 [0.89–1.81]	0.18
LVEF (LVEF ≤ 30 % vs. LVEF > 30 %)	3.28 [1.60–6.71]	0.001	1.85 [1.02–3.35]	0.04	2.02 [1.30–3.14]	0.002
LVEF (LVEF n/m^b^ vs. LVEF > 30 %)	6.58 [4.05–10.7]	<0.0001	5.03 [3.32–7.63]	<0.0001	6.10 [4.27–8.73]	<0.0001
History of hyperlipidemia (yes vs. no)	0.52 [0.34–0.80]	0.003	0.63 [0.44–0.90]	0.01	0.67 [0.49–0.91]	0.01
History of COPD (yes vs. no)	1.89 [0.87–4.13]	0.11	2.26 [1.34–3.81]	0.002	0.83 [0.50–1.39]	0.48
Peak serum CPK level (U/l) (continuous)	1.00 [1.00–1.00]	0.003	1.00 [1.00–1.00]	0.04	1.00 [1.00–1.00]	<0.0001
STEMI (*n* = 1292)	(*n* = 625)		(*n* = 369)		(*n* = 298)	
Sex (women vs. men)	n/a^c^		2.35 [1.06–5.21]	0.04	n/a^c^	
Prior stroke (yes vs. no)	11.2 [2.58–48.7]	0.001	n/a^c^		n/a^c^	
LVEF (LVEF ≤ 30 % vs. LVEF > 30 %)	3.71 [1.01–13.6]	0.05	2.36 [0.74–7.54]	0.15	1.53 [0.58–4.03]	0.39
LVEF (LVEF n/m^b^ vs. LVEF > 30 %)	4.13 [1.22–14.0]	0.02	3.90 [1.30–11.7]	0.02	13.0 [5.72–29.3]	<0.0001
NSTEMI (*n* = 1838)	(*n* = 647)		(*n* = 603)		(*n* = 588)	
Sex (women vs. men)	n/a^c^		1.63 [1.04–2.53]	0.03	n/a^c^	
Prior stroke (yes vs. no)	n/a^c^		n/a^c^		2.15 [1.34–3.46]	0.002
Renal dysfunction (yes vs. no)	2.76 [1.18–6.45]	0.02	1.80 [1.09–2.99]	0.02	n/a^c^	
Prior MI (yes vs. no)	1.81 [1.03–3.18]	0.04	2.04 [1.30–3.21]	0.002	n/a^c^	
LVEF (LVEF ≤ 30 % vs. LVEF > 30 %)	3.08 [1.15–8.22]	0.03	1.88 [0.86–4.13]	0.12	3.19 [1.75–5.80]	0.001
LVEF (LVEF n/m^b^ vs. LVEF > 30 %)	6.24 [3.56–10.9]	<0.0001	5.04 [3.12–8.16]	<0.0001	5.49 [3.54–8.50]	<0.0001
History of hyperlipidemia (yes vs. no)	0.52 [0.32–0.85]	0.01	n/a^c^		0.51 [0.35–0.74]	0.001
History of COPD (yes vs. no)	2.57 [1.05–6.32]	0.04	2.11 [1.16–3.85]	0.01	n/a^c^	
Peak serum CPK level (U/l) (continuous)	1.00 [1.00–1.00]	0.001	n/a^c^		1.00 [1.00–1.00]	0.005
BBB (*n* = 330)	(*n* = 53)		(*n* = 111)		(*n* = 165)	
Prior MI (yes vs. no)	9.18 [1.62–51.9]	0.01^d^	3.17 [1.10–9.12]	0.03	n/a^c^	
Renal dysfunction (yes vs. no)	18.8 [1.04–340.0]	0.05^d^	n/a^c^		n/a^c^	
History of hypertension (yes vs. no)	0.11 [0.02–0.68]	0.02^d^	n/a^c^		n/a^c^	
LVEF (LVEF ≤ 30 % vs. LVEF > 30 %)	N/A^e^		2.41 [0.65–8.99]	0.19	1.14 [0.46–2.83]	0.79
LVEF (LVEF n/m^b^ vs. LVEF > 30 %)	N/A^e^		10.5 [3.27–33.7]	<0.0001	9.44 [3.89–22.9]	<0.0001
Peak serum CPK level (U/l) (continuous	n/a^c^		n/a^c^		1.00 [1.00–1.00]	0.01

The multivariable analysis included as explanatory variables sex, type of AMI (‘dummy’-coded), LVEF (‘dummy’-coded), renal dysfunction, prior stroke, prior MI, history of diabetes, hypertension, hyperlipidemia, angina pectoris and COPD, pre-hospital time, and peak serum CPK level. In the total sample, variables which meet the 0.05 significance level in at least one age category were included and presented. For the sub-samples by type of AMI only the significant factors in that sub-sample model (forward stepwise selection technique) were included and presented

*OR* odds ratio, *CI* confidence interval, *AMI* acute myocardial infarction, *STEMI* ST-elevation myocardial infarction, *NSTEMI* non-ST-elevation myocardial infarction, *BBB* bundle branch block, *LVEF* left ventricular ejection fraction, *COPD* chronic obstructive pulmonary disease, *CPK* creatine phosphokinase

^a^ As 23 patients had no data on peak serum CPK level, the total sample size was 3452 instead of 3475

^b^ n/m, LVEF were not assessed or missing

^c^ n/a, OR and *p* value were not applicable because the analyzed variable did not meet the 0.05 significance level for entry into the model of the sub-sample

^d^ *P* value and OR were assessed in a separate model without the variables regarding LVEF

^e^ N/A, not applicable due to quasi-complete separation of data points detected when ‘LVEF’ was included

### Association between invasive therapy and 28-day-case fatality

In addition to baseline characteristics previously reported, Table 4 shows that the 3 ACs significantly differed from each other in terms of the analyzed outcome and several complications during hospitalization except for ventricular fibrillation, bradycardia, stroke, and any major bleeding complication. The highest 28-day-case fatality of 19.5 % (*n* = 74) was observed in AC 3 receiving conservative therapy and the lowest (2.4 %; *n* = 28) in patients aged 28–65 years treated invasively. In general, 28-day-case fatality was lower in the invasively than non-invasively treated patients in all ACs.

**Table 4:** Clinical complications and outcome by age category and stratified by treatment strategy (*n* = 3475)

	Age category									
Variable	25–64 years			65–74 years			75–84 years			*p* Value
Treatment strategy	All *n* = 1329 (100)	IT *n* = 1187 (89.3)	CT *n* = 142 (10.7)	All *n* = 1083 (100)	IT *n* = 891 (82.3)	CT *n* = 192 (17.7)	All *n* = 1063 (100)	IT *n* = 683 (64.2)	CT *n* = 380 (35.8)	<0.0001
Complications during hospital stay										
Cardiac arrest	76 (5.7)	58 (4.9)	18 (12.7)	110 (10.2)	79 (8.9)	31 (16.2)	176 (16.6)	102 (14.9)	74 (19.5)	<0.0001
Cardiogenic shock	54 (4.1)	48 (4.0)	6 (4.2)	77 (7.1)	66 (7.4)	11 (5.7)	101 (9.5)	75 (11.0)	26 (6.8)	<0.0001
Ventricular fibrillation	32 (2.4)	29 (2.4)	3 (2.1)	32 (3.0)	27 (3.0)	5 (2.6)	31 (2.9)	25 (3.7)	6 (1.6)	0.65
Ventricular tachycardia	109 (8.2)	105 (8.9)	4 (2.8)	66 (6.1)	60 (6.7)	6 (3.1)	54 (5.1)	41 (6.0)	13 (3.4)	0.007
Bradycardia	76 (5.7)	72 (6.1)	4 (2.8)	71 (6.6)	67 (7.5)	4 (2.1)	63 (5.9)	51 (7.5)	12 (3.2)	0.68
Re-infarction	10 (0.8)	10 (0.8)	0	18 (1.7)	13 (1.5)	5 (2.6)	26 (2.5)	20 (2.9)	6 (1.6)	0.04
Stroke	5 (0.4)	5 (0.4)	0	9 (0.8)	7 (0.8)	2 (1.0)	8 (0.8)	5 (0.7)	3 (0.8)	0.32
Pulmonary edema	30 (2.3)	25 (2.1)	5 (3.5)	43 (4.0)	33 (3.7)	10 (5.2)	57 (5.4)	41 (6.0)	16 (4.2)	0.0003
Any major bleeding complication^a^	13 (1.0)	11 (0.9)	2 (1.4)	19 (1.8)	14 (1.6)	5 (2.6)	22 (2.1)	16 (2.3)	6 (1.6)	0.08
Any in-hospital complication (without cardiac arrest)	254 (19.1)	236 (19.9)	18 (12.7)	243 (22.4)	208 (23.3)	35 (18.2)	249 (23.4)	185 (27.1)	64 (16.8)	0.02
Outcome										
28-day-case fatality	44 (3.3)	28 (2.4)	16 (11.3)	74 (6.8)	44 (4.9)	30 (15.6)	138 (13.0)	64 (9.4)	74 (19.5)	<0.0001
Death during hospital stay	43 (3.2)	28 (2.4)	15 (10.6)	74 (6.9)	45 (5.1)	30 (15.6)	146 (13.7)	72 (10.5)	74 (19.5)	<0.0001

Data are presented as number (percentage). *P* values were calculated for comparison of the age categories

*IT* invasive therapy, *CT* conservative therapy

^a^ Major bleeding complication: intracranial or retroperitoneal or any other major spontaneous bleeding

In the multivariable logistic regression analyses, invasive therapy showed a strongly inverse relation with 28-day-case fatality compared with the conservative therapy in all ACs. After adjustment for potential confounding variables the full model (model 2) revealed an OR of 0.35 (95 % CI 0.15–0.84), 0.45 (95 % CI 0.22–0.92), and 0.39 (95 % CI 0.24–0.63) in the AC 1, 2 and 3, respectively (Table 5).

**Table 5:** Association between invasive therapy and 28-day-case fatality by age category

	Age category					
	25–64 years		65–74 years		75–84 years	
	OR [95 % CI]	*p* Value	OR [95 % CI]	*p* Value	OR [95 % CI]	*p* Value
Model 1^a^	0.19 [0.10–0.36]	<0.0001	0.28 [0.17–0.46]	<0.0001	0.43 [0.30–0.62]	<0.0001
Model 2^b^	0.35 [0.15–0.84]	0.02	0.45 [0.22–0.92]	0.03	0.39 [0.24–0.63]	<0.0001

^a^ Model 1 adjusted for sex for the total sample (*n* = 3475)

^b^ Model 2 adjusted for sex, body mass index ≥30 kg/m^2^, type of AMI, renal dysfunction, prior stroke, prior MI, history of diabetes, hypertension, hyperlipidemia, angina pectoris and chronic obstructive pulmonary disease, any in-hospital complication (without cardiac arrest), pre-hospital time, left ventricular ejection fraction, peak serum creatine phosphokinase (CPK) level, and in-hospital medication: dual antiplatelet therapy, beta-blockers, statins, and angiotensin-converting-enzyme inhibitors or angiotensin receptor blockers. As 23 patients had no data on peak serum CPK level, the total sample size was 3452

## Discussion

In the present registry-based study including 3475 consecutively enrolled patients with AMI occurring between 2009 and 2012, we found that several factors such as type of AMI, reduced LVEF, prior stroke, history of no hyperlipidemia, and a low CPK level were strong predictors of a non-invasive therapy independent of age. The sub-group analysis by type of AMI revealed more independent predictors in patients with NSTEMI compared to STEMI. In addition, besides various differences observed between the 3 age groups, we found that an invasive therapy was inversely associated with 28-day-case fatality with almost similar mortality risk reductions in all age groups.

Our findings that NSTEMI and prior stroke were strong predictive factors are in concordance with an earlier study conducted in 1001 elderly STEMI and NSTEMI patients in Germany, which additionally found age, prior MI, renal failure, pre-existing coronary artery disease, Killip Class >II, and supraventricular tachycardia as factors predicting treatment type in patients above 75 years [17]. Our analysis by age groups and type of AMI adds that renal dysfunction and prior MI were significantly associated only in patients presenting with NSTEMI below 75 years. In contrast to our study, where female sex was associated with non-invasive therapy in the total sample above 64 years, Rittger et al. [17] reported that sex had no significant influence on treatment strategy in patients above 75 years. After stratifying the study population by type of AMI, we found that women demonstrated 2-fold higher odds of receiving conservative therapy only in patients aged 65–74 years diagnosed with either STEMI or NSTEMI. Even if other factors such as frailty or severe multimorbidity could have biased sex differences found in observational studies, especially in STEMI patients (see below), one should consider the recently updated clinical practice guideline of the American College of Cardiology and the American Heart Association which highlights that both genders should be treated in the same way [18]. It is known from earlier studies that prevalence of frailty and multiple comorbidities influence the likeliness of receiving invasive therapy and also the outcome of elderly ACS patients [5, 19–21], and were found to be more common in women [21–24]. Therefore, for clinical decision-making and evaluation of the prognosis of elderly ACS patients an assessment tool for end-of-life status, which showed comparable usefulness with clinical risk scores such as the Global Registry of Acute Coronary Events (GRACE) score, might be considered to predict the 1-year all-cause mortality and to select the approximately 8 % of patients with an end-stage illness [22].

In the sub-group of STEMI patients we observed that only a small number did not receive invasive therapy, but this proportion increased by age from 3.4 % in AC 1 to 16.4 % in AC 3. Our multivariable analyses revealed only 3 predictive factors for non-invasive therapy in STEMI patients: LVEF in all ACs, prior stroke in patients below 65 years and female sex in patients aged 65–74 years. ORs of 11 (prior stroke) and 4–13 (LVEF) indicate a strong impact of these factors in STEMI patients. Prior stroke was also reported as predictor of non-invasive therapy in earlier studies regarding STEMI patients [11, 13]. Our study adds that reduced LVEF or heart failure and cardiac enzyme levels such as CPK or troponin might be more important than other factors previously reported. Regarding female sex, a discrepancy between previous reported results exists. Gharacholou et al. [11] reported that female sex was identified as strong factor associated with no reperfusion among the reperfusion-eligible STEMI population analyzed from 226 US hospitals participating in the CRUSADE quality improvement initiative. Another study also reported an association between female sex and not attempting reperfusion in STEMI patients [7]. In contrast, some earlier studies [23, 24] found that being a woman was not an independent predictor in patients presenting with STEMI. As we analyzed factors by age, our study adds the information that female sex might be an independent predictor in patients who were between 65 and 74 years old. However, as mentioned above, missing adjustment for unobserved confounders related with female sex such as frailty, multiple comorbidities or high risk of death could have biased our and previously reported studies. This theory is supported by a recent study in 1104 STEMI patients based on two clinical network registries in Germany which reported that standard of care including performance of primary PCI and procedural success rate were not gender specific, and the adjusted 12-months mortality did not differ between men and women despite significant differences in clinical baseline parameters [25]. However, gender differences in clinical decision-making regarding reperfusion rates and secondary drug treatment prophylaxis were still reported in several countries [26–29]. In contrast to earlier studies in STEMI patients, we did not find diabetes [7, 9, 12], prior MI [9] and delayed presentation (pre-hospital time) [13, 23, 24] as being predictive factors in this sub-group. The comparison across studies is, however, difficult, since we were not able to adjust our analyses for other potential reasons such as patient preference, dementia [13], or contraindications to the use of reperfusion, prior CABG, spontaneous reperfusion [10] or Killip class risk score [10, 17]. In general, we observed that previous reported results vary considerably depending mainly on study design, time period, analyzed factors, proportion of patients not receiving invasive therapy, and country of origin.

Regarding 28-day-case fatality, we observed a clear short-term survival benefit associated with invasive therapy in all 3 age groups after adjustment for various confounding factors including type of AMI, evidence-based medication and in-hospital complications. In contrast to earlier trials [1, 5, 30], we did not observe greater risk reduction from the invasive therapy in patients above 64 years. The ORs found in our study were almost similar, but showed a narrower CI in the eldest group, which strengthens the benefit of an invasive therapy for reperfusion-eligible patients above 74 years. In addition, the occurrence of major bleeding complications was not significantly different between the ACs in our study, but showed a higher rate in the invasively treated patients only in the oldest age group. However, as we did not know the time point of complications’ appearance during hospitalization, we cannot exclude that bleeding complications might have been present before invasive therapy. In summary, our results regarding short-term survival confirmed the previously reported benefit of invasive therapy in AMI patients up to 84 years [5, 31–35]. However, we cannot exclude that the benefit of invasive therapy coexists with a higher risk of major bleeding in patients above 74 years of age as reported previously [1, 5].

### Strength and limitations

Major strength of our study is the setting in a population-based registry with patients consecutively hospitalized with all types of AMI and data collection performed soon after the AMI during the hospital stay. Furthermore, our research covers recent data up to 2012. Despite adjustment for a number of variables, residual confounding cannot be entirely excluded due to further unknown comorbidities or complications such as frailty, cancer and cognitive and physical function, which could have influenced decision to perform an invasive therapy and also short-term mortality. As we do not have information on Killip class, GRACE risk score and comorbid anemia for patients included in this study, we were not able to analyze these potential predictors of clinical decision-making in today’s real-life patient care. In addition, we were not able to address the issue of contraindications or eligibility to the use of invasive procedures, and documented reasons for non-invasive therapy were not assessed in our registry. Finally, our results are limited to AMI- patients who survived at least 24 h after hospitalization and were between 26 and 84 years old.

## Conclusion

In today’s real-life patient care we found that NSTEMI, BBB, prior stroke, reduced LVEF, absence of hyperlipidemia, and low CPK level were the strongest predictors for non-invasive therapy in all age groups. Stratifying the analysis by type of AMI revealed more independent predictors in patients with NSTEMI compared to STEMI. Further research is necessary to investigate the real reasons for non-invasive therapy, especially among elderly women. Moreover, we confirmed that invasive therapy was independently associated with short-term survival benefit regardless of patient’s age.

## Abbreviations

AC, Age category; ACEIs/ARBs, Angiotensin-converting-enzyme inhibitors and/or angiotensin receptor blockers; ACS, Acute coronary syndrome; AMI, Acute myocardial infarction; BBB, Bundle branch block; CABG, Coronary artery bypass grafting; CI, Confidence interval; CPK, Creatine phosphokinase; KORA, Cooperative health research in the region of Augsburg; LVEF, Left ventricular ejection fraction; MI, Myocardial infarction; MONICA, Monitoring trends and determinants in cardiovascular disease; NSTEMI, Non-ST segment elevation myocardial infarction; OR, Odds ratio; PCI, Percutaneous coronary intervention; STEMI, ST-segment elevation myocardial infarction.
